# Basaloid Squamous Cell Carcinoma: An Unusual Ball-Valve Laryngeal Obstruction

**Published:** 2015-05

**Authors:** Sien Hui Tan, Khalid W Hindi, Patricia Ann Chandran, Aun Wee Chong

**Affiliations:** 1 *Department of Otorhinolaryngology, Faculty of Medicine, University Malaya, Kuala Lumpur, Malaysia.*; 2 *Department of Pathology, Faculty of Medicine, University Malaya, Kuala Lumpur, Malaysia.*

**Keywords:** Airway obstruction, Basaloid squamous cell carcinoma, Larynx, Squamous cell carcinoma

## Abstract

**Introduction:**

A rare case of basaloid squamous cell carcinoma (BSCC) of the larynx, which has not been previously reported, is described.

**Case Report:**

A 60-year-old man was presented to the Otolaryngology Department with progressive dyspnoea and dysphagia to solids for over a period of 1 week. Direct laryngoscopy revealed a tumour at the laryngeal aspect of the epiglottis, which prolapsed into the laryngeal inlet each time the patient inspired. This resulted in an inspiratory stridor despite adequate glottic opening and normal mobility of the vocal cords.

**Conclusion:**

Therefore, in cases where a ball-valve lesion causes intermittent life-threatening airway obstruction, BSCC of the larynx, though rare, must be considered as a differential diagnosis.

## Introduction

Basaloid squamous cell carcinoma (BSCC), first described by Wain et al in 1986 (1), is a rare distinct variant of squamous cell carcinoma (SCC). Despite its propensity for the upper aero-digestive tract, BSCC merely comprises less than 1% of laryngeal carcinoma(2). In this paper, a rare case of basaloid squamous cell carcinoma of the larynx, that presented an unusual ball-valve type obstruction of the laryngeal inlet, is described. This unique presentation has not been previously reported in the literature.

## Case Report

A 60-year-old man presented to the Otolaryngology Department with progressive dyspnoea and dysphagia to solids for over a period of 1 week. Further questioning revealed a 6-month history of hoarseness, occasional blood stained sputum, as well as loss of weight and appetite. He had a 20-year smoking history; but denied any alcohol consumption. He had no known medical illness or family history of malignancy.

**Fig1:**
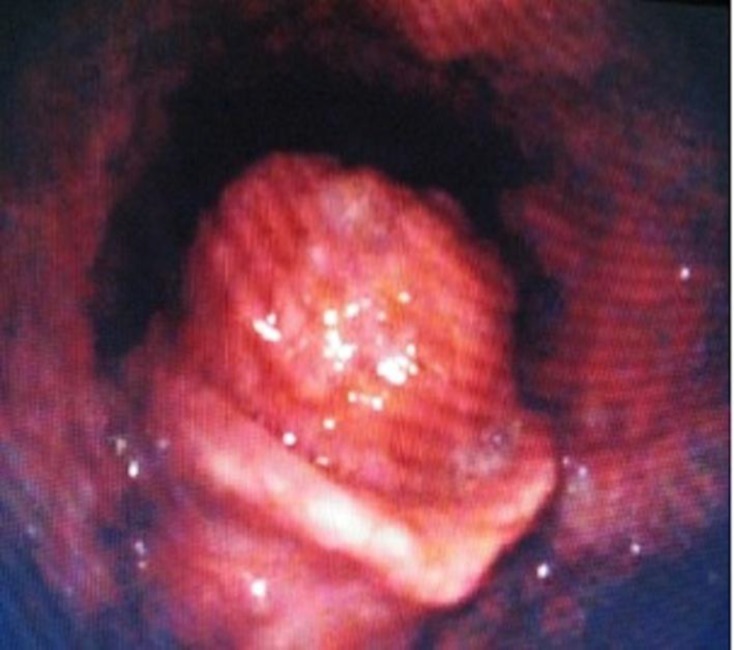
Laryngoscopic view illustrating an exophytic mass at the laryngeal surface of epiglottis causing airway obstruction.

Clinical examination revealed a cachectic looking gentleman with inspiratory stridor. Flexible nasoendoscopy showed an exophytic growth involving the laryngeal surface of the epiglottis, which prolapsed into the laryngeal inlet on inspiration (Fig.1). 

This resulted in a ball-valve type obstruction and prevented visualization of the vocal cords. There were no palpable cervical lymph nodes and the rest of the ear, nose, and throat examinations were unremarkable. 

**Fig 2:**
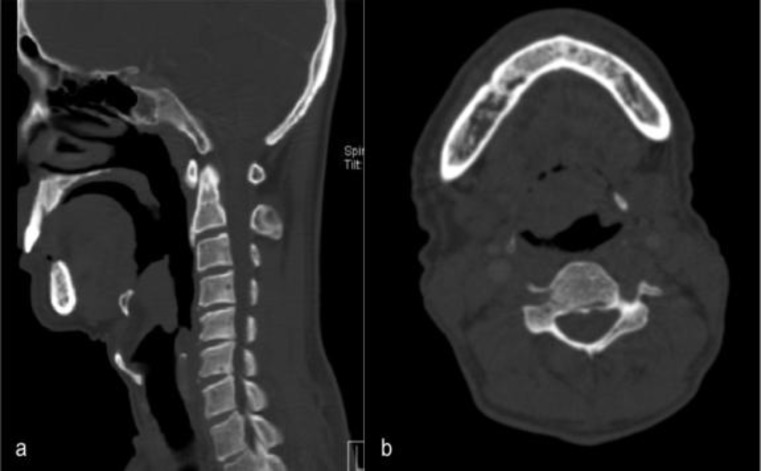
(a) Sagittal CT scan showing an epiglottic mass causing narrowing of the laryngeal inlet.  (b) Axial CT scan showing a large mass at the laryngeal aspect part of the epiglottis.

Computed tomography (CT) of the neck and thorax reported a soft tissue mass in the anterior part of the epiglottis measuring 2.7 x 2.2 x 2.5 cm with extension to the base of the epiglottis, as well as bilateral cervical lymph nodes at level Ib and II, with the largest measuring 1.2 x 0.8 cm (Fig.2).

The patient underwent a tracheostomy under local anesthesia in view of the impending airway obstruction. De-bulking of the tumour was performed and subsequent direct laryngoscopy was carried out to assess the extent of the lesion. The mass was confined to the epiglottis without involvement of the vocal cords, arytenoid cartilages and pyriform fossa. 

**Fig 3:**
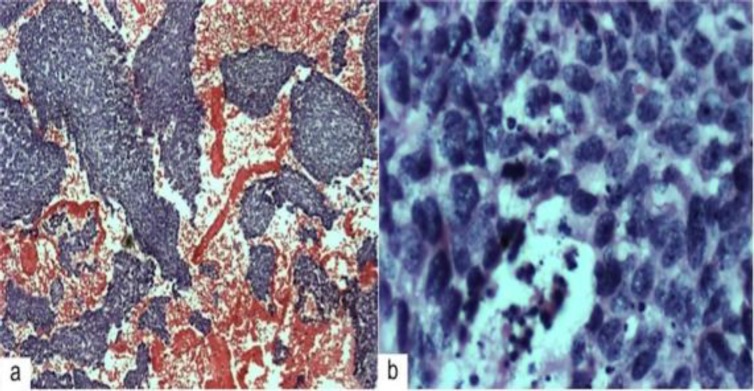
(a) Low power view of the cell block showing clusters of malignant cells with basaloid appearance (Haematoxylin eosin, original magnification x 40). (b) High power magnification showing malignant basaloid cells with coarse chromatin pattern, numerous mitosis, and apoptotic bodies. Some of the cells show intercellular bridges denoting squamous differentiation (Haematoxylin eosin, original magnification x 400).

Microscopy demonstrated islands and clusters of malignant basaloid looking cells with coarse chromatin pattern, numerous mitotic figures, and apoptotic bodies. Some cells showed presence of intercellular bridging denoting squamous differentiation (Fig.3). Immunohistochemically, the cells were positive for epithelial membrane antigen (EMA), neuron-specific enolase (NSE); and negative for chromogranin and synaptophysin. The final histopathological report revealed BSCC.

The patient refused to undergo the proposed supraglottic laryngectomy and neck dissection. Hence, he was referred to the Oncology department for chemoradio- therapy. A year later, there was local recurrence of the tumour and this eventually led to the demise of the patient.

## Discussion

BSCC has a predilection for the head and neck region particularly the supraglottic larynx, the base of the tongue, and the pyriform sinus (3-6); but it has also been described in the esophagus, lung, thymus, anus, cervix, penis, and urinary bladder (7,8). It is believed to originate from a totipotential primitive cell in the basal layer of the surface epithelium or from the proximal ducts of salivary glands (1).

The typical patient with BSCC is an elderly male aged between 60 and 80 years. However, risk factors for BSCC remain undetermined. Soriano et al and Banks et al associated tobacco and alcohol consumption with BSCC patients (6,9); whereas Wieneke et al did not (10). Interestingly, our patient was a chronic smoker but denied any alcohol consumption. Other studies have also linked viral infections such as Epstein-Barr virus (11,12), human papilloma virus, and herpes simplex virus with BSCC.

Clinical presentation of a patient with BSCC in the larynx includes hoarseness, respiratory distress, or dysphagia. Compared to the other described cases in the literature, this patient had extremely atypical symptoms. The tumour at the laryngeal aspect of the epiglottis prolapsed into the laryngeal inlet each time the patient inhaled despite adequate glottic opening and normal mobility of the vocal cords. Consequently, the patient’s inspiratory stridor, though it showed temporary relief of symptoms during coughing, essentially represented recurrent airway obstruction triggered by a ball-valve mechanism between the mass and the laryngeal inlet.

BSCC is defined as an aggressive, high grade, variant of SCC composed of both basaloid and squamous components (13). Basaloid cells have hyperchromatic nuclei and scant cytoplasm, with prominent peripheral palisading and frequent comedo-type necrosis (13). Histological diagnosis can prove to be challenging due to the heterogeneous cellular composition of BSCC and its non-specific macroscopic features. The differential diagnosis of BSCC includes adenoid cystic carcinoma, adenos- quamous carcinoma, and neuroendocrine carcinoma. 

Nevertheless, there are several distinguishing features to differentiate BSCC from the rest. True ducto-glandular differentiation and intracellular mucin are found in adenosquamous carcinoma while adenoid cytic carcinoma contains a myoepithelial component and lacks squamous differentiation (13). Neuroe- ndocrine markers such as chromogranin and synaptophysin help discern BSCC from neuroendocrine carcinoma (13). In this case, both histopathological and immunochemical findings were crucial in obtaining the final diagnosis of BSCC.

Consensus regarding treatment of BSCC has yet to be established. Aggressive multimodality treatment is usually advocated with surgery, followed by radiotherapy and chemotherapy. In cases of locally advanced BSCC of the larynx, Soriano et al proposed an organ conservation approach with initial chemotherapy followed by radiotherapy (6). However, total laryngectomy with neck dissection, due to the increased likelihood of cervical lymph node involvement, is usually preferred. Our patient unfortunately refused surgery and eventually succumbed to local recurrence of the tumour a year later. 

There is an ongoing debate over the prognosis of BSCC compared to conventional SCC. Although some studies have demonstrated similar outcomes in both BSCC and SCC (9,14), other studies believe that BSCC has a worse survival outcome (1,5,15). Fritsch et al proposed that BSCC’s poor prognosis is unrelated to its location within the larynx (15), disease stage, and therapeutic strategy. However, BSCC tends to be present in advanced stages resulting in a poorer global prognosis. The prevalent supraglottic location of these tumours usually causes a delay in the diagnosis, as the tumour has to infiltrate into the vocal cords to produce symptoms. 

## Conclusion

In cases where a ball-valve lesion causes intermittent life-threatening airway obstruction, BSCC of the larynx, though rare, must be considered as a differential diagnosis.

## References

[1] Wain SL, Kier R, Vollmer RT, Bossen EH (1986). Basaloid-squamous carcinoma of the tongue, hypopharynx and larynx: report of 10 cases. Hum Pathol.

[2] Akyol MU, Seckin S, Albayrak L, Ozdem C (1995). Basaloid squamous cell carcinoma of the larynx. Eur Arch Otorhinolaryngol.

[3] Barnes L, Ferlito A, Altavilla G, MacMillan C, Rinaldo A, Doglioni C (1996). Basaloid squamous cell carcinoma of the head and neck: clinicopathologic features and differential diagnosis. Ann Otol Rhinol Laryngol.

[4] Paulino AF, Singh B, Shah JP, Huvos AG (2000). Basaloid squamous cell carcinoma of the head and neck. Laryngoscope.

[5] Bahar G, Feinmesser R, Popovtzer A, Ulanovsky D, Nageris B, Marshak G (2003). Basaloid squamous carcinoma of the larynx. Am J Otolaryngol.

[6] Soriano E, Faure C, Lantuejoul S, Reyt E, Bolla M, Brambilla E (2008). Course and prognosis of basaloid squamous cell carcinoma of the head and neck: a case control study of 62 patients. Eur J Cancer.

[7] Suster S, Rosai J (1991). Thymic carcinoma: a clinicopathologic study of 60 cases. Cancer.

[8] Ereno C, Gaafar A, Garmendia M, Bilbao FJ, López JI (2008). Basaloid squamous cell carcinoma of the head and neck: a clinicopathological and follow-up study of 40 cases and review of the literature. Head Neck Pathol.

[9] Banks ER, Frierson Jr HF, Mills SE, George E, Zarbo RJ, Swanson PE (1992). Basaloid squamous cell carcinoma of the head and neck: a clinicopathologic and immunohistochemical study of 40 cases. Am J Surg Pathol.

[10] Wieneke JA, Thompson LDR, Wenig BM (1999). Basaloid squamous cell carcinoma of the sinonasal tract. Cancer.

[11] Wan SK, Chan JK, Lau WH, Yip TT (1995). Basaloid-squamous carcinoma of the nasopharynx An Epstein-Barr virus-associated neoplasm compared with morphologically identical tumors occurring in other sites. Cancer.

[12] Kleist B, Bankau A, Lorenz G, Jager B, Poetsch M (2004). Different risk factors in basaloid and common head and neck cancer. Laryngoscope.

[13] Cardesa A, Zidar N, Ereno C, Barnes L, Eveson JW, Reichart P, Sidransky D (2005). Basaloid squamous cell carcinoma. Pathology and genetics of head and neck tumours.

[14] Luna MA, El Naggar A, Parichatikanond P, Weber RS, Batsakis JG (1990). Basaloid squamous carcinoma of the upper aerodigestive tract. Cancer.

[15] Fritsch VA, Lentsch EJ (2014). Basaloid squamous cell carcinoma of the larynx: Analysis of 145 cases with comparison to conventional squamous cell carcinoma. Head Neck.

